# Predicting neurological recovery with Canonical Autocorrelation Embeddings

**DOI:** 10.1371/journal.pone.0210966

**Published:** 2019-01-28

**Authors:** Maria De-Arteaga, Jieshi Chen, Peter Huggins, Jonathan Elmer, Gilles Clermont, Artur Dubrawski

**Affiliations:** 1 Machine Learning Department, Carnegie Mellon University, Pittsburgh, United States of America; 2 Heinz College, Carnegie Mellon University, Pittsburgh, United States of America; 3 Auton Lab, School of Computer Science, Carnegie Mellon University, Pittsburgh, United States of America; 4 Department of Emergency Medicine, University of Pittsburgh School of Medicine, Pittsburgh, United States of America; 5 Department of Critical Care Medicine, University of Pittsburgh School of Medicine, Pittsburgh, United States of America; 6 CRISMA laboratory, Department of Critical Care Medicine, University of Pittsburgh School of Medicine, Pittsburgh, United States of America; University of California San Francisco, UNITED STATES

## Abstract

Early prediction of the potential for neurological recovery after resuscitation from cardiac arrest is difficult but important. Currently, no clinical finding or combination of findings are sufficient to accurately predict or preclude favorable recovery of comatose patients in the first 24 to 48 hours after resuscitation. Thus, life-sustaining therapy is often continued for several days in patients whose irrecoverable injury is not yet recognized. Conversely, early withdrawal of life-sustaining therapy increases mortality among patients who otherwise might have gone on to recover. In this work, we present Canonical Autocorrelation Analysis (CAA) and Canonical Autocorrelation Embeddings (CAE), novel methods suitable for identifying complex patterns in high-resolution multivariate data often collected in highly monitored clinical environments such as intensive care units. CAE embeds sets of datapoints onto a space that characterizes their latent correlation structures and allows direct comparison of these structures through the use of a distance metric. The methodology may be particularly suitable when the unit of analysis is not just an individual datapoint but a dataset, as for instance in patients for whom physiological measures are recorded over time, and where changes of correlation patterns in these datasets are informative for the task at hand.

We present a proof of concept to illustrate the potential utility of CAE by applying it to characterize electroencephalographic recordings from 80 comatose survivors of cardiac arrest, aiming to identify patients who will survive to hospital discharge with favorable functional recovery. Our results show that with very low probability of making a Type 1 error, we are able to identify 32.5% of patients who are likely to have a good neurological outcome, some of whom have otherwise unfavorable clinical characteristics. Importantly, some of these had 5% predicted chance of favorable recovery based on initial illness severity measures alone. Providing this information to support clinical decision-making could motivate the continuation of life-sustaining therapies for these patients.

## Introduction

Cardiac arrest is the most common cause of death in high-income nations [1]. In the United States alone, over 350,000 people suffer out-of-hospital cardiac arrest each year [2]. Despite advances in care, only a minority of those that are resuscitated and survive to hospital admission are discharged alive, and even fewer enjoy a favorable neurological recovery [2, 3]. Among non-survivors, the most common proximate cause of death is withdrawal of life-sustaining therapy based on perceived poor neurological prognosis [3, 4]. This decision may be motivated by the rarity of favorable recovery, the emotional and financial hardship placed on families faced with the prospect of even a few days of intensive care, or fear of survival with severe disability.

Unfortunately, accurate neurological prognostication after cardiac arrest is challenging, particularly in the first 3 to 5 days after resuscitation [5]. Life-sustaining therapy is still often withdrawn before prognosis is certain, unnecessarily reducing rates of favorable recovery [3, 6, 7, 8]. At the same time, patients with brain injury that is ultimately deemed irrecoverable are often supported for days while providers gather sufficient data to make such an assessment.

**Fig 1:**
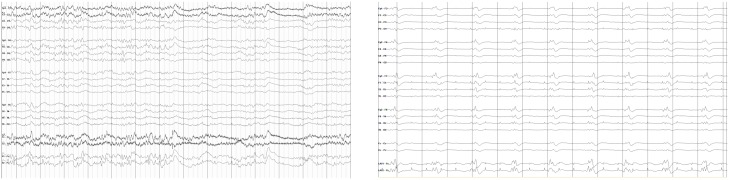
**(Left)** EEG of a post-arrest patient who goes on to recover. **(Right)** EEG of a patient with poor neurological prognosis.

Multiple modalities which might inform early prognostication have been explored [9, 10, 11, 12]. Of particular interest is the rich electroecephalographic (EEG) data that may be obtained. Research indicates that EEG signals can improve prediction accuracy [10, 13, 14]. Qualitatively, some EEG patterns such as seizures suggest severe brain injury [15]. Quantitatively, patterns with strong correlations between channels or over time are suggestive of diffuse cortical damage seen after non-survivable brain injury [14, 16]. Within EEG, as in many biological systems, entropy is a marker of information content [17]. By contrast, strong spatial or temporal correlations are an ominous predictor of severe brain injury [10, 14, 16]. Because these correlations may be subtle and/or complex, they may be inapparent to providers qualitatively interpreting the EEG, leading to growing interest in quantitative EEG analysis. Fig 1 shows an example of an EEG of a post-arrest patient with mild brain injury who goes on to enjoy a favorable recovery and an example of an EEG of a patient with severe brain injury, for which correlations across channels are very strong. Motivated by this, our goal is to characterize patients in terms of their multivariate, non-linear structures of correlation and use the resulting featurization to identify patients who likely have the potential for favorable neurological recovery.

We propose Canonical Autocorrelation Analysis (CAA) as a method for automated discovery of multiple-to-multiple correlation structures within a set of features. Through the introduction of a distance metric between CAA correlation structures, we are able to define Canonical Autocorrelation Embeddings (CAE), a feature space embedding in which each individual/object is represented by the set of its multivariate correlation structures. In this feature space embedding, traditional machine learning algorithms that rely on distance metrics, such as nonparametric clustering and k-nearest neighbors (k-nn), can be applied to compare correlation structures.

This methodology is particularly fitting to tasks where multiple potentially correlated data points are recorded over space or time for each individual or unit of study. For example, in clinical medicine several vital signs or other physiological measures may be repeatedly sampled for each patient being monitored. Because physiological processes are interdependent and interact, analyzing the correlation structure between several such processes may reveal otherwise unrecognized patterns that may characterize the current state of the patient [16, 18]. In this work, we demonstrate the utility of CAE by presenting a specific clinical example: predicting future neurological recovery in a cohort of comatose survivors of cardiac arrest using sets of quantitative EEG autocorrelations.

Because of the difficulty identifying patients with potential for recovery and desire to limit futile care described above, patients may be at risk for withdrawal of life-sustaining therapy when their potential to recover goes unrecognized [3]. To reduce this risk, we propose a decision-support system that provides recommendations to the clinician whenever it is confident that a patient is likely to have a positive neurological recovery and defers in all other instances. Therefore, rather than always providing a recommendation, the algorithm only does so when it is confident life-sustaining therapies should be continued, a prediction it reaches based on patterns in multivariate correlation structures of the EEG that the clinician might not have observed. In all other cases the algorithm will defer to the clinician’s judgment.

In the remainder of this paper, Section 1 presents a brief review of related work. Section 2.1 discusses the task and data in more detail. In Section 2.1, CAA and CAE are introduced, as well as the use of a k-nn algorithm in the resulting embedded space. Section 3 contains the experimental results, Section 4 discusses our findings and Section 5 summarizes the conclusions and future work.

## 1 Related work

Canonical Correlation Analysis (CCA) is a statistical method first introduced by [19], useful for exploring relationships between two sets of variables. It is used in machine learning, with applications to medicine, biology and finance, e.g., [20, 21, 22, 23]. Sparse CCA, an *ℓ*_1_ variant of CCA, was proposed by [23, 24]. This method adds constraints to guarantee sparse solutions, which limits the number of features being correlated. Given two matrices $X\in R^{n\times p}$ and $Y\in R^{n\times q}$, CCA aims to find linear combinations of their columns that maximize the correlation between them. Usually, *X* and *Y* are two disjoint matrix representations for one set of objects, so that each matrix is using a strictly different set of variables to describe them. Assuming *X* and *Y* have been standardized, the constrained optimization problem is shown in Eq 1. When *c*_1_ and *c*_2_ are small, solutions will be sparse and thus only a few features are correlated.

$$\begin{array}{rr} & max_{u,v}u^{T}X^{T}Yv\\ {\left|\left|u\right|\right|}_{2}^{2}\leq 1,{\left|\left|v\right|\right|}_{2}^{2}\leq 1 & {\left|\left|u\right|\right|}_{1}\leq c_{1},{\left|\left|v\right|\right|}_{1}\leq c_{2}\\ \text{for} & 0\leq c_{1}\leq 1,0\leq c_{2}\leq 1 \end{array}$$ (1)

The extension of Sparse CCA for discovery of multivariate correlations within a single set of features to study brain imaging has been previously explored in [20, 21]. Using the notion of autocorrelation, the authors attempt to find underlying components of functional magnetic resonance imaging (fMRI) and EEG, respectively, that have maximum autocorrelation. The types of data used in these works are ordered, both temporally and spatially.

**Fig 2:**
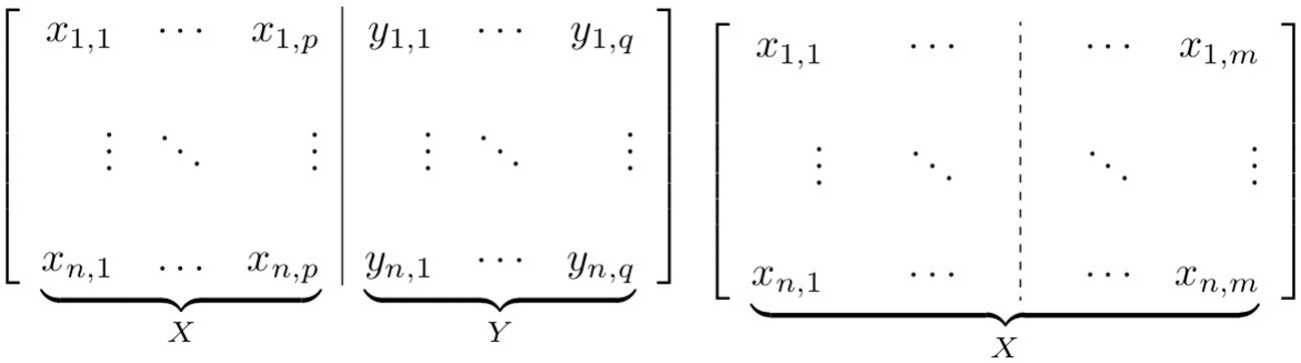
Comparison between scenarios where Sparse CCA and CAA can be used. **(Left)** Sparse CCA finds sparse multiple-to-multiple linear correlations between subsets of the features in matrix *X* and subsets of features in matrix *Y*. **(Right)** CAA extends this to cases where it is not known a priori how to group the features into two sets.

Canonical Autocorrelation Analysis (CAA), the methodology we propose, is a generalized approach to discovering multiple-to-multiple correlations within a set of features. Fig 2 illustrates the different use cases of Sparse CCA and CAA. The proposed formulation also allows for the user to select sets within which correlations are forbidden, which is useful when trivial correlations should be avoided. Moreover, we introduce a distance metric between canonical autocorrelation structures, which gives substantially more power to CAA-based methodology, making it useful for various learning tasks, such as clustering and classification on datasets and distributions.

Other methods for finding sparse representations of data comprised in a single matrix include the well-known Sparse Principal Component Analysis (Sparse PCA). While CAA resembles Sparse PCA in the sense that it finds sparse representations of data contained in one matrix, Sparse PCA maximizes retained *variance* of data in one-dimensional projections, while CAA finds two-dimensional projections where *correlation* across two subsets of features is maximized. CAA specifically seeks projections composed by pairs of strongly correlated linear combinations of features, enabling discovery of hidden characteristic correlations in data, which cannot be easily found with other methods such as Sparse PCA. S2 File explores the difference between the two methods in more detail and from a theoretical perspective.

Extraction of informative projections has been tackled in the past [25, 26]. Our work differs from the existing methodology in two primary ways. First, each of the CAA projection axes is defined by a linear combination of features, rather than a single feature, which helps discover complex structures if they exist. Secondly, rather than finding projections where classes are well-separated, the proposed methodology is unsupervised and it is aimed at characterizing objects or individuals that have a batch of data points associated to them, yielding an embedding where standard machine learning methodologies can be used with minor modifications. In that sense, the extracted projections are different both in their form and in their purpose.

The comparison of correlation structures and principal components has been explored in the literature for decades. Most prominently, [27] discusses comparison of principal components between groups. To do so, they propose a metric inspired by the concept of congruence coefficient [28], which corresponds to the cosine of the angle between the two p-dimensional vectors. Also related to our task is [29], where a metric between covariance matrices is proposed. The notion of a distance metric between canonical autocorrelation structures differs from these because CAA finds a factorization of the correlation matrix where each portion of the correlation matrix is expressed as the outer product of a pair of orthonormal vectors, which define a bi-dimensional space in which the projected data follows a linear correlation. Section 2.3 discusses the proposed metric.

Learning to defer has been studied in the literature as a means to effectively combine algorithmic and human decision-making [30, 31]. When decision-makers are knowledgeable domain experts, as is the case of clinicians providing care to comatose survivors of cardiac arrest, it is desirable to provide a framework in which the algorithm only provides suggestions when confident, and defers to the human in all other cases. Our work incorporates a deferral notion, and the proposed system only provides recommendations for a subset of cases where it is confident of its predictions.

## 2 Methods

### 2.1 Data sources

This study was approved by the University of Pittsburgh Institutional Review Board with a waiver of informed consent. The data used in this case study are derived from 451 comatose survivors of cardiac arrest treated at a single academic medical center between 2010 and 2015 [10, 32]. For each patient, raw EEG data (recorded at 256Hz across 20 electrode channels distributed in space across the scalp) were signal-processed using commercially available FDA-approved software (Persyst(R) Version 12, Persyst Development Corp, Prescott AZ), using standard clinical signal processing engines. The resulting quantitative EEG (qEEG) measures were summarized at a resolution of 1Hz and are available for continuous EEG recordings averaging about 36 hours per patient. The total number of qEEG features is 66 and include seizure probability, amplitude-integrated EEG for the left and right hemispheres of the brain, epileptiform spike detections, suppression ratio, summary frequency measures, and other metrics physicians find informative. The raw EEG data were not available. The full list of features can be found in S1 Table.

**Fig 3:**
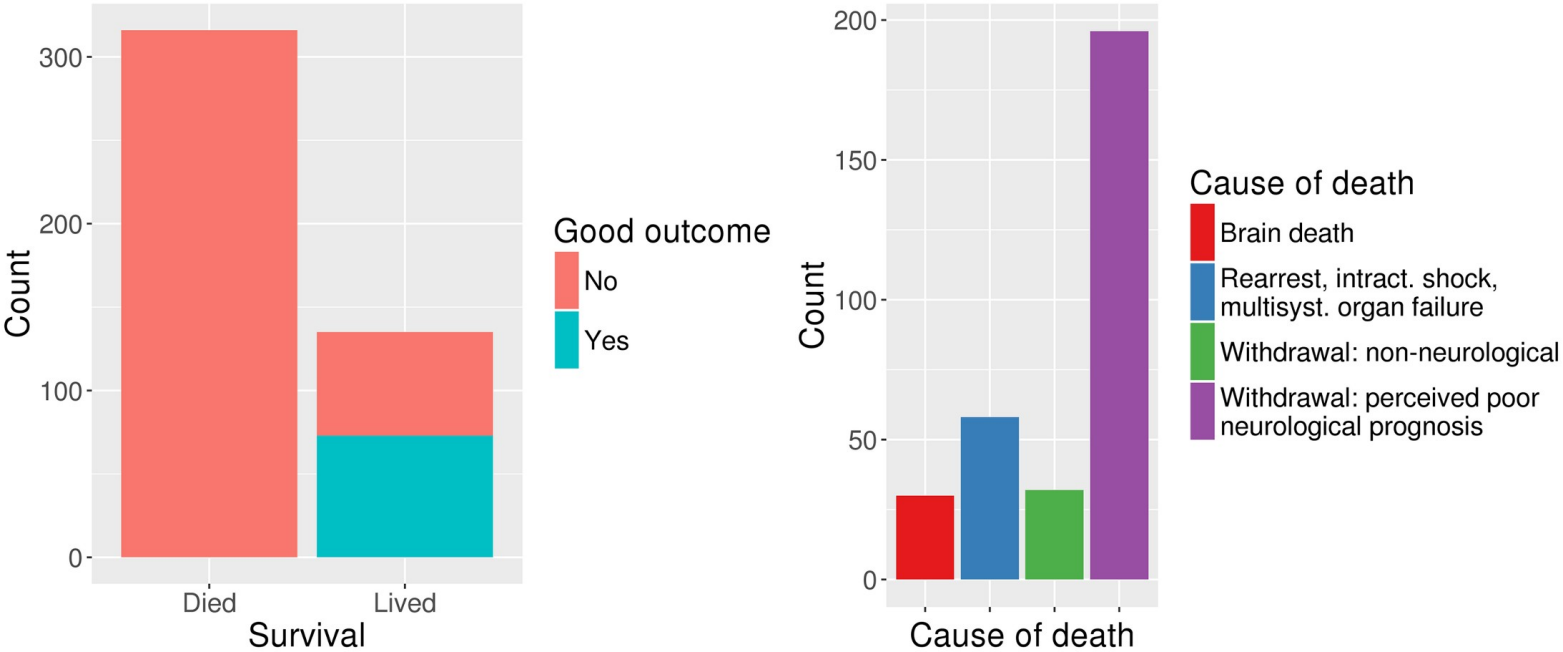
Patient labels indicating survival, outcome and cause of death. **(Left)** Survival and outcome. **(Right)** Cause of death.

Also available for each patient are time-invariant clinical characteristics and outcomes, including survival to hospital discharge. For those who lived, the quality of their functional recovery at discharge was measured using two standard outcome scales: Cerebral Performance Category and modified Rankin Scale. We considered “favorable recovery” to be either a Cerebral Performance Category of 1 or 2 or a modified Rankin Scale score of 0-2 at hospital discharge. For those who died, the proximate cause of death is known. Fig 3 shows this information in detail. The data used in our experiments is limited to patients who survived hospital discharge and who were monitored for at least 36 hours, which corresponds to a total of 80 patients, half of whom had a favorable recovery. Table 1 includes the demographic and clinical characteristics of this cohort. The reasons for limiting our analysis to this subset are explained more in detail in Section 3.

**Table 1:** Demographic and clinical characteristics of cohort of patients considered in the study.

Characteristic	Overall cohort (n = 80)	Favorable outcome (n = 40)	Unfavorable outcome (n = 40)
Age (years)	56 ± 17	51 ± 16	61 ± 14
Gender (F = 1)	23 (29%)	8 (20%)	15 (38%)
Out-of-hospital arrest	61 (76%)	35 (88%)	26 (65%)
Shockable initial rhythm	36 (45%)	24 (60%)	12 (30%)
Pittsburgh Cardiac Arrest Category			
*ii*	42 (53%)	63 (25%)	17 (43%)
*iii*	19 (24%)	6 (15%)	13 (33%)
*iv*	19 (24%)	9 (23%)	10 (25%)
Cardiac etiology	55 (69%))	21 (53%))	34 (85%))
Cardiac catheterization	33 (41%))	22 (55%))	11 (28%))
Temperature management			
33°C	67 (84%))	35 (88%))	32 (80%))
36°C	9 (11%))	4 (10%))	5 (13%))
None	4 (5%)	1 (3%))	3 (8%))
Hospital length of stay (days)	20 [13–27]	16 [11–24]	21 [17–30]

### 2.2 Canonical Autocorrelation Analysis

The goal of CAA is to find multivariate sparse correlations within a single set of variables. In the Sparse CCA framework, this could be understood as having identical matrices *X* and *Y*. Applying Sparse CCA when *X* = *Y* results in solutions *u* = *v*, corresponding to Sparse PCA solutions for *X* [24]. We overcome this issue by introducing a penalty for overlapping feature support. The resulting optimization problem for CAA is shown in Eq 2.

$$\begin{array}{rr} & max_{u,v}u^{T}X^{T}Xv\\ & s.t.\\ {\left|\left|u\right|\right|}_{2}^{2}\leq 1,{\left|\left|v\right|\right|}_{2}^{2}\leq 1 & {\left|\left|u\right|\right|}_{1}\leq c_{1},{\left|\left|v\right|\right|}_{1}\leq c_{2}\\ & \sum_{i=1}^{m}\left|u_{i}v_{i}\right|=0\\ \text{for} & 0\leq c_{1}\leq 1,0\leq c_{2}\leq 1 \end{array}$$ (2)

This can be understood as a new generalization of the Penalized Matrix Decomposition [24]. Note that the equality constraint in Eq 2 can be seen as a weighted *L*_1_ penalty when either *u* or *v* are fixed. Replacing the equality constraint by an inequality constraint gives a biconvex problem, while resulting in the same solution. Therefore, we can solve it through alternate convex search [33], as shown in Algorithm 1.

**Algorithm 1**: CAA via alternate convex search

**1** Initialize *v* s.t. ||*v*||_2_ = 1;

**2** **repeat**

**3**  $u\leftarrow \underset{u}{\text{arg}\;\text{max}}\;u^{T}X^{T}Xv$

**4**  s.t. ${\left||u|\right|}_{2}^{2}\leq 1$, ||*u*||_1_ ≤ *c*_1_, $\sum_{i=1}^{m}|u_{i}\left|\right|v_{i}|=0$

**5**  $v\leftarrow \underset{v}{\text{arg}\;\text{max}}\;u^{T}X^{T}Xv$

**6**  s.t. ${\left||v|\right|}_{2}^{2}\leq 1$, ||*v*||_1_ ≤ *c*_1_, $\sum_{i=1}^{m}|u_{i}\left|\right|v_{i}|=0$

**7** **until** *u*, *v* *converge*;

**8** *d* ← *u*^*T*^*X*^*T*^*Xv*;

At each iteration, the resulting convex problem can be solved through the Karush-Kuhn-Tucker (KKT) conditions. The pseudo-code for solving the convex problems at each iteration of the alternate convex search is provided in Algorithm 2, where we solve for *u* without loss of generality. For a detailed derivation see S1 File.

**Algorithm 2**: CAA alternate convex search iteration via KKT conditions

**1** $\lambda_{1}=\max_{i}\frac{|{\left(X^{T}Xv\right)}_{i}|}{|v_{i}|}$;

**2** **if** $\left|\right|\frac{S_{\Phi (v\lambda_{1},0)}\left(X^{T}Xv\right)}{\left|\right|S_{\Phi (v\lambda_{1},0)}\left(X^{T}Xv\right){\left|\right|}_{2}^{2}}{\left|\right|}_{1}\leq c_{1}$ **then**

**3**  **return** $u=\frac{S_{\Phi (v\lambda_{1},0)}\left(X^{T}Xv\right)}{\left|\right|S_{\Phi (v\lambda_{1},0)}\left(X^{T}Xv\right){\left|\right|}_{2}^{2}}$

**4** **else**

**5**  Binary search to find λ_2_ s.t. $\left|\right|\frac{S_{\Phi (v\lambda_{1},\lambda_{2})}\left(X^{T}Xv\right)}{\left|\right|S_{\Phi (v\lambda_{1},\lambda_{2})}\left(X^{T}Xv\right){\left|\right|}_{2}^{2}}{\left|\right|}_{1}=c_{1}$;

**6**  **return** $u=\frac{S_{\Phi (v\lambda_{1},\lambda_{2})}\left(X^{T}Xv\right)}{\left|\right|S_{\Phi (v\lambda_{1},\lambda_{2})}\left(X^{T}Xv\right){\left|\right|}_{2}^{2}}$

**7** **end**

To find multiple pairs of CAA canonical vectors, Algorithm 1 can be repeated iteratively, replacing *X*^*T*^*X* with a matrix from which the already found correlations are removed, as shown in Eq 3, where *d* = *u*^*T*^*X*^*T*^*Xv*.

$$\begin{array}{c} X^{T}X-d\left(uv^{T}+vu^{T}\right) \end{array}$$ (3)

In order to enable the discovery of non-linear correlations by extending the feature space with subsequent powers of the original features [34], we modify the optimization problem to extend the concept of disjoint support to sets of features. This also prevents the discovered correlations to be dominated by relationships between features that are already known to be correlated by design. Assuming each feature *x*_*i*_ has a subset *S*_*i*_ of associated indices of other features that should not be included as correlates of *x*_*i*_, the resulting optimization problem follows Eq 4.

$$\begin{array}{rr} & max_{u,v}u^{T}X^{T}Xv\\ {\left|\left|u\right|\right|}_{2}^{2}\leq 1,{\left|\left|v\right|\right|}_{2}^{2}\leq 1 & {\left|\left|u\right|\right|}_{1}\leq c_{1},{\left|\left|v\right|\right|}_{1}\leq c_{2}\\ & \sum_{i=1}^{m}\sum_{j\in S_{i}}\left|u_{i}v_{i}\right|=0\\ \text{for} & 0\leq c_{1}\leq 1,0\leq c_{2}\leq 1 \end{array}$$ (4)

The new constraint for disjoint support can still be understood as a weighted-L_1_ penalty at each iteration of the biconvex optimization algorithm. Hence, the problem can still be solved as discussed above, with the only difference that the parameters of the soft-thresholding operator will change.

### 2.3 Canonical Autocorrelation Embeddings

CAA allows us to find bi-dimensional projections where the data closely follows a linear distribution. Each axis of these projections corresponds to a linear combination of the original features, and their respective coefficients are represented in a pair of vectors $u,v\in R^{m}$. We call each pair *u*, *v* a CAA canonical space, and each CAA model may consist of one or more of such canonical spaces.

Since the correlations discovered by CAA are defined by pairs of vectors in $R^{m}$, we can measure the distance between two CAA canonical spaces in terms of Euler angles defining the rotation from one pair of axes to the other. Measuring the angle between two vectors is equivalent to measuring the arc between them, and ||*u*||_2_ = ||*v*||_2_ = 1 ∀*i*, therefore, the distance between two CAA canonical spaces *C*_1_ and *C*_2_ can be defined as shown in Eq 5. This yields an embedding that we refer to as Canonical Autocorrelation Embedding (CAE).

$$\begin{array}{c} d\left(C_{1},C_{2}\right)=\min(||u_{1}-u_{2}{\left|\right|}_{2}+\left|\right|v_{1}-v_{2}{\left|\right|}_{2}\;,\;||u_{1}-v_{2}{\left|\right|}_{2}+\left|\right|v_{1}-u_{2}{\left|\right|}_{2}) \end{array}$$ (5)

It is easy to show that this metric satisfies the necessary conditions for a well-defined distance, see S3 File for the proof.

Even though we believe that Eq 5 provides a good distance metric that captures what we desire to measure, we do not claim this is the only nor necessarily the best such metric, and it is appropriate to continue exploring alternatives. S4 File contains a short discussion of why “principal angles”, a metric that is commonly used to measure distance between subspaces and which naturally comes to mind in this setting, is actually not well-suited in this case.

### 2.4 K-Nearest correlations

Having formulated a distance metric between pairs of CAA canonical spaces enables us to employ a range of distance-based machine learning algorithms, such as k-means, hierarchical clustering, or k-nn, to leverage similarities among correlation structures present in data. One additional complexity in our case is that each subset of data being compared may be represented by more than one CAA canonical space, and therefore more than one point in the embedding.

This setting can be incorporated into the k-nn framework by calculating the class probability for each correlation structure through the votes of their *k* nearest neighbors, and then aggregating over all correlations associated to an object using log-odds, as shown in Eq 6, where *n*_*p*,*i*,*j*_ denotes the class label of the j*th* neighbor of the i*th* correlation of patient *p*.

$$\begin{array}{c} \begin{array}{c} q_{i}=\frac{\sum_{j=1}^{k}n_{p,i,j}}{k}\\ {\hat{y}}_{p}=\text{log}\left(\prod_{i=1}^{m_{p}}\frac{q_{i}}{1-q_{i}}\right) \end{array} \end{array}$$ (6)

However, it is likely that some type of correlation structures will be common to both classes, while others are discriminative. To reduce noise and allow for those discriminative correlations to lead the decision, we incorporate a threshold *t*, so that log-odds are only calculated over those correlation structures with a class probability that is discriminative enough, as shown in Eq 7. Incorporating this threshold also enhances interpretability of the comparisons, as it reduces the number of structures that are used for making a prediction, making it easier for practitioners to understand which correlations appear relevant for the task at hand. The parameters *k*, indicating the number of neighbors, and *t* can be tuned through cross-validation.

$$\begin{array}{c} \begin{array}{c} {\hat{y}}_{p}=\text{log}\left(\prod_{i=1}^{m_{p}}I_{\left(|q_{i}-0.5|>t\right)}\frac{q_{i}}{1-q_{i}}\right) \end{array} \end{array}$$ (7)

## 3 Results

Our principal goal is to help improve care given to comatose survivors of cardiac arrest through a decision support system that can boost the accuracy and timeliness of prognostication. To do so, we propose a new way to characterize patients using their latent multivariate correlation structures, and use the resulting featurization of data to build predictive models. The results presented in this section leverage data collected over a five year period at an academic medical center to provide a proof of concept of the proposed methodology.

As seen in Fig 3, the main cause of death for this patient population is withdrawal of life-sustaining therapy due to perceived poor neurological prognosis. However, as mentioned in Section 1, it is possible that in some cases treatment might be withdrawn too early, a decision which nearly invariably leads to death and precludes favorable recovery. Including those patients in our training set could result in the model replicating mistakes clinicians may be making, leading to a self-fulfilling prophecy. Considering this and the fact that our goal is to predict positive neurological outcome rather than survival alone, we train our model using only those patients who lived, making our target label whether they had a good or a poor neurological outcome. This also reduces the risk for unaccounted treatment effects, since presumably all patients who are kept on live support receive a minimum standard of appropriate care, while therapeutic nihilism may influence outcomes for patients for whom life-supporting therapies are interrupted.

**Fig 4:**
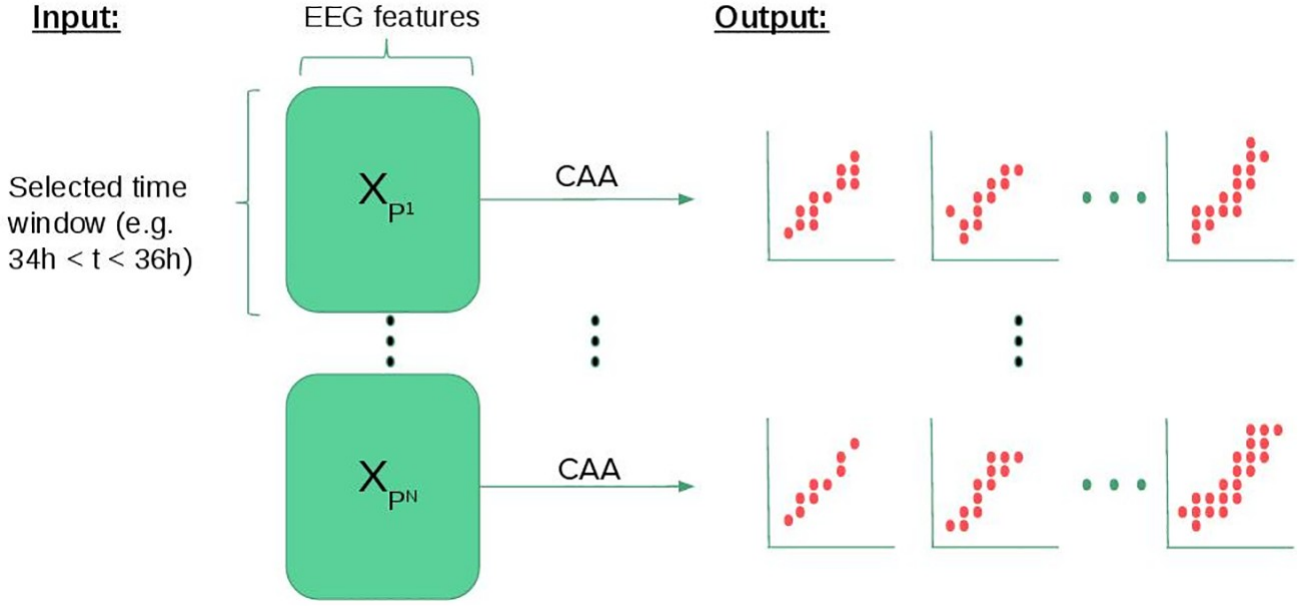
Diagram illustrating CAA patient characterization using EEG features as inputs.

For each patient, their entire qEEG record is available, with lengths varying from less than an hour to more than a week. We aim to predict recovery as early as possible, but the earlier we attempt prediction, the more challenging it is. For the purposes of this experiment, we target prediction after 36 hours of monitoring. We use CAA to characterize a two hour epoch between hours 34 and 36. We choose 36 hours because we are interested in a period where the EEG is relatively static so that we do not need to account for temporal trends within the analyzed epoch. Clinically, patients are cooled down for 24 hours then allowed to rewarm at about 0.25-0.5C/hr. Both temperature and medications used to suppress shivering can alter the EEG [10]. At 36 hours, patients are back to a normal body temperature. The specific question the proposed model answers is: can the correlations present during this epoch predict whether the patient will go on to enjoy a favorable recovery? We consider only two hours because it can be expected that each patient’s state fluctuates over time, and the resulting variance could obfuscate important patterns of correlation. Identifying temporal trends, or inferring meta-correlation structures that describe these trends, is an important subject of future work beyond the scope of current analysis. Fig 4 illustrates the process of characterization of multiple patients’ EEG data with CAA.

**Fig 5:**
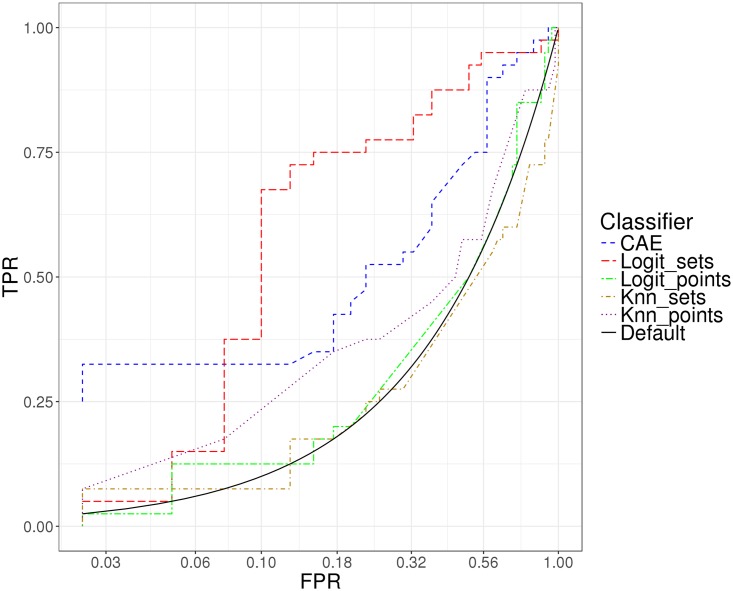
ROC curves showing performance of CAE, logistic regression on sets, logistic regression on points, k-nn on sets and k-nn on points. X-axis in log-scale to emphasize low FPR region.

**Fig 6:**
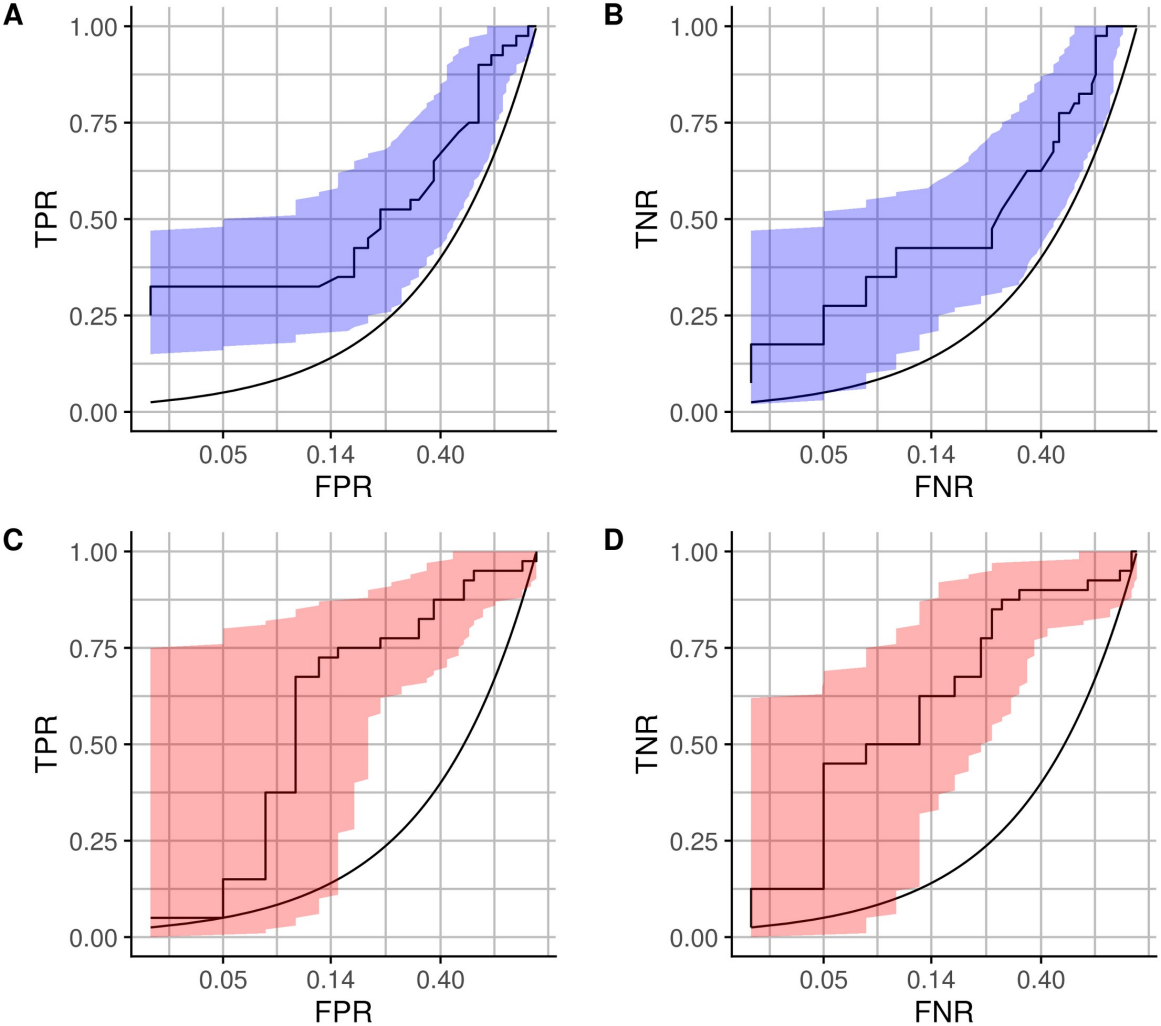
ROC curves with 95% confidence intervals for CAE (AUC = 0.71 with 95% confidence interval of [0.6, 0.82]) and logistic regression on sets (AUC = 0.81 with 95% confidence interval of [0.71, 0.91]), x-axis in log-scale. **(A)** CAE, TPR vs. FPR. **(B)** CAE, TNR vs. FNR. **(C)** Logit on sets, TPR vs. FPR. **(D)** Logit on sets, TNR vs. FNR.

In order to avoid spurious results, we only consider CAA canonical projections that yield correlations with *R*^2^ > 0.25. Moreover, to ensure that only reasonably close neighbors are used for matching, we prune connections by only considering distances smaller than $\sqrt{2}$, a threshold that corresponds to a 90° rotation over one axis. Empirical results of k-Nearest Correlations with CAE obtained through 10-fold cross-validation, with tuning parameters *k* and *t* in an internal 10-fold cross-validation loop within each training fold, are presented in Figs 5 and 6.

For baseline comparison, we use a popular approach: extract features based on metrics calculated over windows of time and apply standard classifiers to the resulting featurization [35, 36, 37]. We calculate quartiles for each input feature over two hours preceding the 36-hour mark, and provide them as features to logistic regression with lasso regularization [38] and k-nn with Euclidean distance. We refer to these as *logistic regression on sets* and *k-nn on sets*, respectively. To emphasize the importance of considering a window of time rather than a snapshot, we also compare against the same two algorithms taking as input the last data point after 36 hours of monitoring, that is, the recording at one time step. We refer to this approach as *logistic regression on points* and *k-nn on points*, respectively. The parameters are chosen through 10-fold cross-validation. The results are included in Figs 5 and 6.

Finally, we apply the resulting system to those patients who were withdrawn from life-sustaining therapies. Amongst 31 patients who received life-sustaining therapies for at least 36 hours before withdrawal of life-sustaining therapies, five patients would have been marked by our classifier as very likely to recover at a threshold of FPR equal to 0.025.

## 4 Discussion

Recall that the proposed decision-support system is one that only makes recommendations when it has strong indications that the patient is likely to have a positive neurological recovery, and defers in all other cases. We evaluate the performance of the algorithm at the thresholds at which it would make recommendations, which we characterize through low false positive rates (FPR). The true positive rate (TPR) at a given FPR indicates what portion of positives–TPR—would be retrieved while assuring that no more than a given rate of negatives–FPR—will be incorrectly labeled as positive.

Due to the gravity of errors in this scenario, the tolerance for false positives should be extremely low. Receiver Operator Curves (ROC) shown in Figs 5 and 6 display true positive rates at different false positive rates, with the x-axis in log-scale to emphasize the low FPR region. While Area Under the Curve (AUC) is reported, it is important to note that this performance metric is not particularly relevant in our case (nor in any other case in which there is a fixed threshold at which decisions are made). AUC is a measure that allows us to aggregate performance over all possible FPR thresholds, but what we really care about is the performance at the thresholds that are chosen for deployment.

The results presented in Fig 5 show that the proposed methodology has predictive power, and the comparison to k-nn using Euclidean distance highlights the role of CAE. While the performance of all other methods at low FPR is no better than random, the performance of CAE at low FPR is promising, with a TPR of 0.325 and corresponding 95% confidence interval [0.125, 0.46] at a FPR of 0.025 (Fig 6A). This means that with very low probability of making a Type I error, we are able to confidently identify at least 12.5% of the patients who will go on to have a positive neurological recovery. If the deployment setup changed, an ensemble model including CAE and logistic regression could be used to draw benefits from both of its components: high recall at low FPR of CAE, and overall good separability between outcome classes of logistic regression.

Even though consensus guidelines advocate maintaining life-sustaining therapies for at least 72 hours after cardiac arrest [7, 8], the burden associated to continuing life-support for patients who will not have a positive neurological recovery still often leads clinicians to withdraw treatment earlier [3]. Thus, the ability of CAE to confidently identify patients that will likely recover with a good outcome has the potential to save lives.

To appropriately estimate the potential impact of such a decision support system in terms of lives saved, it is useful to compare against physicians’ assessments to validate if the predictions made with the proposed approach are non-redundant to what doctors already know. Each patient in our dataset is classified by Pittsburgh Cardiac Arrest Category, a 4-level, validated prognostic indicator assigned in the first six hours of their stay [39]. This classification indicates whether the patient is awake with little brain injury (category *i*), in a mild to moderate brain injury with good heart and lung function (category *ii*), in a mild to moderate brain injury but poor heart and/or lung activity (category *iii*), or severe brain injury with loss of some brainstem reflexes (category *iv*). While patients in category *i* have an associated probability of survival of 80%, and 60% probability of having a positive neurological recovery, patients in category *iv* have probabilities of 10%, and 5%, respectively. At a FPR lower than 0.025, the proposed methodology correctly identified a category *iv* patient who later went on to have a positive recovery. This constitutes a preliminary indication that the patterns of correlations in neurological activity measured with EEG constitute novel findings and have the potential to improve reliability of prognostication.

As discussed in Section 3, amongst those patients whose cause of death is withdrawal of life-sustaining therapy for perceived neurological prognosis, 5 out of 31 patients who received life-sustaining therapies for at least 36 hours would have been marked by our system as likely to have a positive recovery. Two of these patients had received a Pittsburgh Cardiac Arrest Category of *iv*. The remaining three received an initial Pittsburgh Cardiac Arrest Category of *ii*. While we do not have ground truth regarding counterfactuals of what would have happened if life-sustaining therapies had been continued for these patients, these results provide further indication that CAE is not simply leveraging patterns that are already being used by physicians.

While it would also be desirable to identify patients who have a very small probability of neurological recovery, we note that neither of the models would be able to provide confident recommendations to withdraw life-sustaining therapies while guaranteeing low false negative rates (FNR). Fig 6B and 6D show the results for CAE and logit on sets at low FNR. These negative results may in part be explained by the fact that the available labeled data encodes positive/negative outcomes, but these are not limited to just neurological activity. A patient could have a positive neurological recovery but have other medical complications that limit function and thus result in a *bad outcome* label. Meanwhile, the positive recovery label is sure to indicate positive neurological recovery (as well as positive recovery in other areas).

A research direction that could further improve the performance of CAE is correlation trajectory modeling. While our model captures correlations observed within an interval of time, and in that sense it goes beyond a purely stationary approach, leveraging the sequential structures in data and using all data collected during a patients’ stay is desirable. Methodologically, this calls for the development of models for trajectory modeling of multivariate correlation structures. This could also encompass further exploration of additional distance metrics that could incorporate other types of information. By leveraging more information, such an approach would have the potential of providing earlier and more specific predictions.

An additional direction for performance enhancement comes from the fact that our characterization of brain activity with CAA is motivated by the importance clinicians place on correlations. However, the correlations they know to be informative are across raw EEG channel measurements, and it is likely that at the current level of data aggregation, a big portion of the information may be to some extent obfuscated. This does not constitute a risk in terms of the validity of the results presented in this paper, but it means that further promising results may be expected from characterizing correlations in raw EEG signals. Such models could also lead to biological insights that may not be easily derived with the current approach.

An important challenge that arises in this setting is the selective labels problem [40]. Selective labels is a common yet understudied problem that occurs whenever historical decision-making blinds us to the true outcome for certain instances. In the case of predicting neurological recovery, we may only observe the true outcome when the clinicians decide to extend life sustaining therapy, while we are blind to the conterfactual of what would have happened in those cases for which life sustaining therapy is withdrawn early. If patients for whom treatment was stopped early are significantly different from those for whom it was not, which is possibly the case, machine learning models trained only on the observed outcomes might have a lower-than-desired performance for that group.

## 5 Conclusions and future work

Cardiac arrest is a leading cause of death around the world, coma after cardiac arrest is common, and good neurological recovery is rare. Everyday, clinicians are tasked with making a prediction that determines whether they will continue life-sustaining therapies for their patients in coma or not. Motivated by the emphasis the clinicians place on potential informativeness of the correlation structures in EEG data, we have proposed a way to characterize and compare patients based on the latent structures of multivariate correlations, and use such information to predict positive neurological recovery. To do so, we have proposed a new formulation of Canonical Autocorrelation Analysis (CAA), a method that automatically finds subsets of features of data that form strong multiple-to-multiple correlations. We have also introduced Canonical Autocorrelation Embeddings (CAE) to enable the comparison of discovered correlation structures. CAE makes powerful and well established machine learning methodologies that rely on the use of distance metrics applicable to the task at hand.

The results presented in this paper constitute a proof of concept. Future work involves collecting more data to train and validate the model. It is reasonable to believe that there may be a substantial heterogeneity across patients, hence experiments using more data of more subjects are a necessary next step. Applying CAE to the raw EEG channels rather than to the aggregated featurizations of data would also be interesting to explore once that data becomes available.

In addition, we are developing machine learning methodology to tackle the selective labeling problem by incorporating clinicians’ domain knowledge while not reproducing their mistakes. Developing proper evaluation metrics to assess performance under selective labels, and finding ways to tackle the blindness that may result from this problem, is an important ingredient needed to successfully use machine learning to save lives in clinical settings.

## Supporting information

S1 Table qEEG features. Complete list of qEEG features available and used in this study.
(PDF)

S1 File CAA optimization. Solution of CAA optimization problem via KKT conditions.
(PDF)

S2 File CAA and Sparse PCA. Discussion of the relationship and differences between CAA and Sparse PCA.
(PDF)

S3 File CAA distance metric. Proof of CAA well-defined distance metric.
(PDF)

S4 File Principal angles and CAA. Discussion of why principal angles are not a well suited distance for CAA canonical spaces.
(PDF)
