# Supplementary material for: Predicting neurological recovery with Canonical Autocorrelation Embeddings
Source: PLoS One. 2019 Jan 28;14(1):e0210966. doi: 10.1371/journal.pone.0210966 (PMC6349311; doi:10.1371/journal.pone.0210966)
Supplement: S1 Table — Complete list of qEEG features available and used in this study. (PDF) [file pone.0210966.s001.pdf]

S1 Table. qEEG features. Complete list of qEEG features available and used in this study.

Feature	Details
Artifact Intensity	Muscle
Artifact Intensity	Chew
Artifact Intensity	V-Eye
Artifact Intensity	L-Eye
Artifact Detector (Signal quality -Electrode 1)	
Artifact Detector (Signal quality -Electrode 2)	
Artifact Detector (Signal quality -Electrode 3)	
Artifact Detector (Signal quality -Electrode 4)	
Artifact Detector (Signal quality -Electrode 5)	
Artifact Detector (Signal quality -Electrode 6)	
Artifact Detector (Signal quality -Electrode 7)	
Artifact Detector (Signal quality -Electrode 8)	
Artifact Detector (Signal quality -Electrode 9)	
Artifact Detector (Signal quality -Electrode 10)	
Artifact Detector (Signal quality -Electrode 11)	
Artifact Detector (Signal quality -Electrode 12)	
Artifact Detector (Signal quality -Electrode 13)	
Artifact Detector (Signal quality -Electrode 14)	
Artifact Detector (Signal quality -Electrode 15)	
Artifact Detector (Signal quality -Electrode 16)	
Artifact Detector (Signal quality -Electrode 17)	
Artifact Detector (Signal quality -Electrode 18)	
Seizure Probability	
aEEG, Left Hemisphere	Max
aEEG, Left Hemisphere	Min
aEEG, Left Hemisphere	Median
aEEG, Left Hemisphere	Q75%
aEEG, Left Hemisphere	Q25%
aEEG, Right Hemisphere	Max
aEEG, Right Hemisphere	Min
aEEG, Right Hemisphere	Median
aEEG, Right Hemisphere	Q75%
aEEG, Right Hemisphere	Q25%
aEEG+(filt)(LFF0.16sec,HFF(off),custom aEEG2-20 512),Left Hem	Max
aEEG+(filt)(LFF0.16sec,HFF(off),custom aEEG2-20 512),Left Hem	Min
aEEG+(filt)(LFF0.16sec,HFF(off),custom aEEG2-20 512),Left Hem	Median

aEEG+(filt)(LFF0.16sec,HFF(off),custom 512),Left Hem	aEEG2-20	Q75%
aEEG+(filt)(LFF0.16sec,HFF(off),custom 512),Left Hem	aEEG2-20	Q25%
aEEG+(filt)(LFF0.16sec,HFF(off),custom 512),Right Hem	aEEG2-20	Max
aEEG+(filt)(LFF0.16sec,HFF(off),custom 512),Right Hem	aEEG2-20	Min
aEEG+(filt)(LFF0.16sec,HFF(off),custom 512),Right Hem	aEEG2-20	Median
aEEG+(filt)(LFF0.16sec,HFF(off),custom 512),Right Hem	aEEG2-20	Q75%
aEEG+(filt)(LFF0.16sec,HFF(off),custom 512),Right Hem	aEEG2-20	Q25%
PeakEnvelope, 1 - 20 Hz, Left Hemisphere		
PeakEnvelope, 1 - 20 Hz, Right Hemisphere		
Spike Detections		
Suppression Ratio, Left Hemisphere		
Suppression Ratio, Right Hemisphere		
FFT Power, 1 - 4 Hz, Left Hemisphere		
FFT Power, 1 - 4 Hz, Right Hemisphere		
FFT Power, 4 - 8 Hz, Left Hemisphere		
FFT Power, 4 - 8 Hz, Right Hemisphere		
FFT Power, 8 - 13 Hz, Left Hemisphere		
FFT Power, 8 - 13 Hz, Right Hemisphere		
FFT Power, 13 - 20 Hz, Left Hemisphere		
FFT Power, 13 - 20 Hz, Right Hemisphere		
FFT Alpha/Delta, 8-13/1-4 Hz, Left Hemisphere		
FFT Alpha/Delta, 8-13/1-4 Hz, Right Hemisphere		
Rhythmicity Spectrogram, Left Hemisphere		1-4Hz
Rhythmicity Spectrogram, Left Hemisphere		4-8Hz
Rhythmicity Spectrogram, Left Hemisphere		8-13Hz
Rhythmicity Spectrogram, Left Hemisphere		13-20Hz
Rhythmicity Spectrogram, Right Hemisphere		1-4Hz
Rhythmicity Spectrogram, Right Hemisphere		4-8Hz
Rhythmicity Spectrogram, Right Hemisphere		8-13Hz
Rhythmicity Spectrogram, Right Hemisphere		13-20Hz
