# Supplementary material for: Predicting neurological recovery with Canonical Autocorrelation Embeddings
Source: PLoS One. 2019 Jan 28;14(1):e0210966. doi: 10.1371/journal.pone.0210966 (PMC6349311; doi:10.1371/journal.pone.0210966)
Supplement: S1 File — Solution of CAA optimization problem via KKT conditions. (PDF) [file pone.0210966.s002.pdf]

S1 File. CAA optimization. Solution of CAA optimization problem via KKT conditions.

Without loss of generality, assuming v is fixed and we are optimizing for u, the optimization problem in Lagrangian form can be written as formulated in Eq. 1.

$$\min_u -u^T X^T X v + \sum_{i=1}^{m} (\lambda_1 ||v_i||_1 + \lambda_2)||u_i||_1 + \lambda_3 ||u||_2^2 - \lambda_2 c_1 - \lambda_3 \quad (1)$$

$$\text{for} \quad 0 \leq c_1 \leq 1, 0 \leq c_2 \leq 10 \leq \lambda_1, 0 \leq \lambda_2, 0 \leq \lambda_3$$

The KKT conditions are:

- **Stationarity:** $0 \in -X^T X v + 2\lambda_3 u + \Gamma$
  for $\Gamma_i = (\lambda_2 + \lambda_1 |v_i|) sgn(u_i)\ \forall i = 1, ..., m$
- **Complementary slackness:**
  $\lambda_1 \sum_{i=1}^{m} |u_i||v_i| = 0$; $\lambda_2(||u||_1^2 - c_1) = 0$; $\lambda_3(||u||_2^2 - 1) = 0$
- **Primal feasibility:**
  $\sum_{i=1}^{m} |u_i||v_i| \leq 0$; $||u||_1^2 - c_1 \leq 0$; $||u||_2^2 - 1 \leq 0$
- **Dual feasibility:** $0 \leq \lambda_i$, $i = 1, 2, 3$

From complementary slackness and primal feasibility, either $\lambda_3 = 0$ and $||u||_2 \leq 1$, or $\lambda_3 > 0$ and $||u||_2 = 1$. Assuming $\lambda_3 > 0$ and solving the stationarity condition, we obtain that for $i = 1, ..., m$, Eq. 2 holds, where $S_\lambda(x)$ is the soft-thresholding operator.

$$2\lambda_3 u_i = S_{(\lambda_1 |v_i| + \lambda_2)}((X^T X v)_i) \quad (2)$$

From complementary slackness, λ_3 must be such that $||u||_2 = 1$, therefore, Eq. 3 is obtained.

$$u = \frac{S_{\Phi(v)}(X^T X v)}{||S_{\Phi(v)}(X^T X v)||_2^2} \quad (3)$$

$$\begin{aligned} \Phi(v, \lambda_1, \lambda_2) : \mathbb{R}^m &\longrightarrow \mathbb{R}^m \\ v_i &\longrightarrow \lambda_1 |v_i| + \lambda_2 \end{aligned}$$

Additionally, λ_1 must be such that $\sum_{i=1}^{m} |u_i||v_i| = 0$, which will be guaranteed by setting $\lambda_1 = \max_i \frac{|(X^T X v)_i|}{|v_i|}$. Finally, either $\lambda_2 = 0$ results in a feasible solution, or λ_2 is chosen such that $||u||_1 = c_1$, which can be done through a binary search.
