# Supplementary material for: Predicting neurological recovery with Canonical Autocorrelation Embeddings
Source: PLoS One. 2019 Jan 28;14(1):e0210966. doi: 10.1371/journal.pone.0210966 (PMC6349311; doi:10.1371/journal.pone.0210966)
Supplement: S2 File — Discussion of the relationship and differences between CAA and Sparse PCA. (PDF) [file pone.0210966.s003.pdf]

S2 File. CAA and Sparse PCA. Discussion of the relationship and differences between CAA and Sparse PCA.

As mentioned in Section 2, CAA and Sparse PCA have fundamentally different objectives, but given that Sparse CCA applied to identical matrices ($X = Y$) results in Sparse PCA components, it is worth taking a look at the details of how CAA and Sparse PCA differ. While Sparse PCA finds one-dimensional projections of data that maximize *variance* of data, CAA finds two-dimensional projections where *correlation* between the two sets is maximized. Furthermore, it is easy to see how the variables retrieved by CAA differ from those retrieved by Sparse PCA. As previously mentioned, applying Sparse CCA to matrices $X = Y$ results in Sparse PCA solutions $u = v$ (Witten et al. (2009)). Therefore, Sparse PCA can be written as

$$\begin{aligned} & max_{u,v} u^T X^T X v \\ ||u||_2^2 \leq 1, ||v||_2^2 \leq 1 \quad & ||u||_1 \leq c_1, ||v||_1 \leq c_2 \end{aligned} \tag{1}$$

In the following, we analyze how the objective values retrieved by CAA (Eq.2) and Sparse PCA (Eq. 1) differ.

- Sparse PCA optimal criterion value retrieved:

$$\begin{aligned} u^T X^T X u &= (\textstyle\sum_{i \in P} u_i X_i)^T (\textstyle\sum_{j \in P} u_j X_j) \\ &= \sum_{i \in P} \sum_{j \in P} u_i u_j X_i^T X_j \\ \text{for } P = \{i|u_i \neq 0\} & \end{aligned}$$

- CAA optimal criterion value retrieved:

$$u^T X^T X v = (\sum_{i \in P_1} u_i X_i)^T (\sum_{i \in P_2} v_i X_i)$$
$$\text{for } P_1 = \{i|u_i \neq 0\},\ P_2 = \{i|v_i \neq 0\}$$

Accounting for the constraint that the vectors u, v in the solution are orthogonal, we can rewrite this as:

$$\begin{aligned} & (\sum_{i \in P_1} u_i X_i)^T (\sum_{j \in P_2} v_j X_j) \text{ s.t. } P_1 \cap P_2 = \emptyset \\ = \; & (\sum_{i \in P_1} \sum_{j \in P_2} u_i v_j X_i^T X_j) \text{ s.t. } P_1 \cap P_2 = \emptyset. \end{aligned}$$

Notice that in the case of Sparse PCA, all interactions between variables in the subset P are considered, while CAA only considers interactions across two disjoint groups. Therefore, there are two types of interactions Sparse PCA considers that CAA does not: the variance of each variable and correlation/covariance between variables in the same subset. Note the first one is only relevant for Sparse PCA when using the covariance matrix, but the second is relevant both when Sparse PCA is applied to the correlation matrix or to the covariance matrix. As a result, CAA and Sparse PCA optimize different objectives, retrieving vectors that involve different subsets of features, and such subsets correspond to different types of structures in data.
