# Supplementary material for: Predicting neurological recovery with Canonical Autocorrelation Embeddings
Source: PLoS One. 2019 Jan 28;14(1):e0210966. doi: 10.1371/journal.pone.0210966 (PMC6349311; doi:10.1371/journal.pone.0210966)
Supplement: S3 File — Proof of CAA well-defined distance metric. (PDF) [file pone.0210966.s004.pdf]

S3 File. CAA distance metric. Proof of CAA well-defined distance metric.

In this File we prove that the metric defined to measure the distance between CAA canonical spaces satisfies the necessary conditions to be a well-defined distance.

$$d(C_1, C_2) = \min(||u_1 - u_2||_2 + ||v_1 - v_2||_2 \ , \ ||u_1 - v_2||_2 + ||v_1 - u_2||_2) \quad (1)$$

- Non-negativity: stems directly from the non-negativity of the ℓ_2 norm, together with the fact that the set of non-negative real numbers is closed under the summation and minimum operations.
- Identity:

$$\begin{aligned} 0 &= \min(||u_1 - u_2||_2 + ||v_1 - v_2||_2 \ , \ ||u_1 - v_2||_2 + ||v_1 - u_2||_2) \\ \Leftrightarrow 0 &= ||u_1 - u_2||_2 + ||v_1 - v_2||_2 \ \vee \ 0 = ||u_1 - v_2||_2 + ||v_1 - u_2||_2 \\ &\Leftrightarrow (0 = ||u_1 - u_2||_2 \wedge 0 = ||v_1 - v_2||_2) \\ &\vee \ (0 = ||u_1 - v_2||_2 \wedge 0 = ||v_1 - u_2||_2) \\ &\Leftrightarrow (u_1 = u_2 \wedge v_1 = v_2) \\ &\vee \ (u_1 = v_2 \wedge v_1 = u_2) \end{aligned}$$

  Given that we are dealing with these as non-ordered pairs, $d(C_1, C_2) = 0 \Leftrightarrow C_1 = C_2$.

- Symmetry: Stems directly from the fact that we define C_1 and C_2 as non-ordered pairs, hence the definition of the distance for each is exactly the same.
- Triangle inequality: The triangle inequality comes as a result of the triangle inequality of the ℓ_2 norm. We want to show that

$$d(C_1, C_3) \leq d(C_1, C_2) + d(C_2, C_3)$$

$$\begin{aligned} d(C_1, C_3) &\leq ||u_1 - u_3||_2 + ||v_1 - v_3||_2 \\ &= ||u_1 - u_3 + u_2 - u_2||_2 + ||v_1 - v_3 + v_2 - v_2||_2 \\ &\leq ||u_1 - u_2||_2 + ||u_2 - u_3||_2 + ||v_1 - v_2||_2 + ||v_2 - v_3||_2 \\ &= ||u_1 - u_2||_2 + ||v_1 - v_2||_2 + ||u_2 - u_3||_2 + ||v_2 - v_3||_2 \end{aligned}$$

  Through an analogous process,

$$\begin{aligned} d(C_1, C_3) &\leq ||u_1 - u_3 + v_2 - v_2||_2 + ||v_1 - v_3 + u_2 - u_2||_2 \\ &\leq ||u_1 - v_2||_2 + ||v_1 - u_2||_2 + ||v_2 - u_3||_2 + ||u_2 - v_3||_2 \end{aligned}$$

  Additionally, the following is also true:

$$d(C_1, C_3) \leq ||u_1 - v_3||_2 + ||v_1 - u_3||_2$$

  Therefore, through analogous reasoning, we derive the following two sets of inequalities:

$$
\begin{aligned}
d(C_1, C_3) &\leq ||u_1 - v_3 + u_2 - u_2||_2 + ||v_1 - u_3 + v_2 - v_2||_2 \\
&\leq ||u_1 - u_2||_2 + ||v_1 - v_2||_2 + ||u_2 - v_3||_2 + ||v_2 - u_3||_2
\end{aligned}
$$

$$
\begin{aligned}
d(C_1, C_3) &\leq ||u_1 - v_3 + v_2 - v_2||_2 + ||v_1 - u_3 + u_2 - u_2||_2 \\
&\leq ||u_1 - v_2||_2 + ||v_1 - u_2||_2 + ||v_2 - v_3||_2 + ||u_2 - u_3||_2
\end{aligned}
$$

The four inequalities we have derived span the four possible cases for $d(C_1, C_2) + d(C_2, C_3)$, which concludes our proof.
