# Supplementary material for: Predicting neurological recovery with Canonical Autocorrelation Embeddings
Source: PLoS One. 2019 Jan 28;14(1):e0210966. doi: 10.1371/journal.pone.0210966 (PMC6349311; doi:10.1371/journal.pone.0210966)
Supplement: S4 File — Discussion of why principal angles are not a well suited distance for CAA canonical spaces. (PDF) [file pone.0210966.s005.pdf]

S4 File. Principal angles and CAA. Discussion of why principal angles are not a well-suited distance for CAA canonical spaces.

Although principal angles might initially seem like a good alternative to measure distances between CAA canonical spaces, note that this is not a viable option. Even though each pair of vectors defining a CAA canonical space constitute an orthonormal basis of a subspace, two orthogonal basis defining the same subspace do not represent the same correlation structure. This can be derived from the fact that, as shown in Section 3.2.2, two different pairs of vectors cannot represent the same correlation structure. It is also easy to understand why this would not be the case with a simple counterexample in $\mathbb{R}^3$. Consider the following two pairs of vectors:

$$\begin{cases} u_1 = (1, 0, 0) \\ v_1 = (0, 1, 0) \end{cases} \qquad \begin{cases} u_2 = (\frac{1}{\sqrt{2}}, \frac{1}{\sqrt{2}}, 0) \\ v_2 = (\frac{1}{\sqrt{2}}, -\frac{1}{\sqrt{2}}, 0) \end{cases}$$

Even though they are both orthonormal bases of the same subspace, $u_1 v_1^T \neq u_2 v_2^T$.
